# Renal and Vascular Mechanisms of Thiazolidinedione-Induced Fluid Retention

**DOI:** 10.1155/2008/943614

**Published:** 2008-09-07

**Authors:** Tianxin Yang, Sunhapas Soodvilai

**Affiliations:** Department of Internal Medicine, University of Utah and Veterans Affairs Medical Center, Salt Lake City, UT 84132-2412, USA

## Abstract

Thiazolidinediones (TZDs) are peroxisome proliferator-activated receptor subtype *γ* (PPAR*γ*) activators that are clinically used as an insulin sensitizer for glycemic control in patients with type 2 diabetes. Additionally, TZDs exhibit novel anti-inflammatory, antioxidant, and antiproliferative properties, indicating therapeutic potential for a wide variety of diseases associated with diabetes and other conditions. The clinical applications of TZDs are limited by the common major side effect of fluid retention. A better understanding of the molecular mechanism of TZD-induced fluid retention is essential for the development of novel therapies with improved safety profiles. An important breakthrough in the field is the finding that the renal collecting duct is a major site for increased fluid reabsorption in response to rosiglitazone or pioglitazone. New evidence also indicates that increased vascular permeability in adipose tissues may contribute to edema formation and body weight gain. Future research should therefore be directed at achieving a better understanding of the detailed mechanisms of TZD-induced increases in renal sodium transport and in vascular permeability.

## 1. INTRODUCTION

Thiazolidinediones (TZDs), such as rosiglitazone and pioglitazone, are highly effective for the treatment of type 2 diabetes and are widely prescribed. Unfortunately, fluid retention has emerged as the most common and serious side effect of TZDs and has become the most frequent cause of discontinuation of therapy. The incidence of TZD-induced fluid retention ranges from 7% in monotherapy and to as high as 15% when combined with insulin [1–3]. The fluid retention is often presented as peripheral edema, which can progress into pulmonary edema and congestive heart failure. TZD use leads to a 6-7% increase in blood volume in healthy volunteers [4, 5]. This blood volume expansion can dilute the red blood cell concentration, producing a reduced hematocrit. In fact, changes in hematocrit have been used as a surrogate marker for TZD-induced plasma volume expansion. The fluid retention is often resistant to loop diuretics but is reversed by withdrawing the drug. Many aspects of TZD-induced fluid retention have been covered by excellent review articles [6–12]. This review will emphasize renal sodium retention and vascular hyperpermeability as prominent mechanisms of TZD-induced fluid retention. We will also introduce several possible treatment strategies.

## 2. RENAL MECHANISM

The kidney is the key regulator of electrolyte balance and water conservation. Fluid retention at the renal level is suggested by evidence that TZD-induced edema is associated with reduced urinary sodium and water excretion. Song et al. reported that chronic three-day administration of rosiglitazone to Sprague Dawley rats significantly reduced urine volume (by 22%) and sodium excretion (by 44%) [13]. These findings lead us to speculate that renal mechanisms play a major role in TZD-induced fluid retention. TZDs may cause renal fluid reabsorption directly by affecting tubular transport, renal sodium retention, and vascular hyperpermeability or indirectly by affecting renal hemodynamics or processes. Yang et al. examined the effect of a PPAR*γ* agonist, GI262570 (farglitazar), on the glomerular filtration rate, effective renal plasma flow, and renal filtration fraction in chronically catheter-implanted conscious rats [14]. In this study, glomerular filtration rate was determined by using fluorescein isothiocyanate (FITC)-inulin and renal blood flow by using para-aminohippurate (PAH). A 10-day infusion of GI262570 decreased hematocrit, hemoglobin, and serum albumin (all *P* < .05), indicating volume expansion, but did not alter glomerular filtration rate, effective renal plasma flow, or renal filtration fraction. This indicates that PPAR*γ* agonist-induced volume expansion is not related to changes in renal hemodynamics [14]. This observation is reinforced by a human study in which the six-week administration of pioglitazone to healthy volunteers led to sodium retention without a significant effect on glomerular filtration rate or renal blood flow [15]. This lack of change in renal hemodynamics is, however, not universally reported. The three-day administration of rosiglitazone in Sprague Dawley rates induced a 35% reduction in creatinine clearance, an indirect measure of the glomerular filtration rate [13]. It is unclear whether or not this discrepancy is related to differences in glomerular filtration rate measurement techniques or other experimental protocols.

The lack of solid evidence to support the alteration of renal hemodynamic parameters following treatment with PPAR*γ* ligands suggests the possibility of a direct influence on tubular transport processes. The regulation of NaCl reabsorption in the kidney can occur at the level of sodium transport proteins lining the renal epithelia. These sodium transporters include basolateral Na-K-ATPase, and the following apical transporters that vary with individual nephron segments: the sodium hydrogenexchanger subtype III (NHE3) and the sodium phosphate cotransporter subtype II (NaPi-2) in the proximal convoluted tubule, the bumetanide-sensitive Na-K-2Cl cotransporter (NKCC2 or BSC1) in the thick ascending limb, the thiazide-sensitive Na-Cl cotransporter (NCC or TSC) in the distal convoluted tubule, and the amiloride-sensitive sodium channel (ENaC) in the collecting duct. The major water channel proteins (aquaporins, AQPs) in the kidney include AQP1-4, of which AQP1 and AQP2 function on the apical membrane, and AQP3 and AQP4 on the basolateral membrane [16]. The study of Song et al. is the first to provide a comprehensive examination of the effects of PPAR*γ* agonists on various renal sodium and water transport proteins [13]. In that study, a three-day rosiglitazone treatment increasedthe whole kidney protein level of the *α*-1 subunit of Na-K-ATPase, NKCC2, NHE3, AQP2, and AQP3 [13]. These findings suggest that increases in sodium transport may occur in the proximal convoluted tubule and the thick ascending limb.

The collecting duct reabsorbs approximately 2-3% of the filtered sodium load primarily through ENaC, which is comprised of three subunits, *α*, *β*, and *γ*. These proteins are vital to day-to-day adjustment of sodium reabsorption and are regulated by the hormones aldosterone and insulin [17–19]. A key mediator of aldosterone activation of ENaC is serum and glucocorticoid regulated kinase 1 (SGK1) [20, 21]. Activated SGK1 prevents ENaC degradation by inactivating the ubiquitin ligase Nedd4-2 [22]. Nedd4-2 interacts with the PY motif of ENaC leading to endocytosis and degradation of the channel [22]. Prior to the conditional knockout (KO) studies, three major lines of evidence indicated that the activation of sodium transport processes in the distal nephron may underlie TZD-induced fluid retention. First, within the kidney, PPAR*γ* is highly expressed in the renal medullary collecting duct, with lower expression levels in glomeruli, proximal tubules, and microvasculature. This was demonstrated by both RT-PCR and microdissection as well as by in situ hybridization techniques [23–25]. Second, in a cultured human cortical collecting duct (CCD) cell line, PPAR*γ* agonists increased levels of cell surface *α*-ENaC. This is paralleled by an increase in SGK1 mRNA, which is abolished by pretreatment with a specific PPAR*γ* antagonist, leading to increased levels of cell surface *α*-ENaC. Electrophoretic mobility shift assays further suggest that these effects are caused by the binding of PPAR*γ* to a specific response element in the SGK1 promoter [20]. Third, in vivo evidence shows that GI262570 stimulates sodium and water reabsorption from the distal nephron in Sprague Dawley rats [26]. This evidence comes from increases in plasma sodium and chloride concentrations with concomitant decreases in plasma potassium concentration. Reciprocal changes in plasma NaCl and potassium levels are typically seen as a consequence of renal mineralocorticoid activation promoting NaCl reabsorption and potassium secretion in the distal nephron [26]. Additionally, mRNA levels for a group of genes involved in distal nephron sodium and water absorption in the kidney medulla are changed with GI262570 treatment [26].

**Figure 1:**
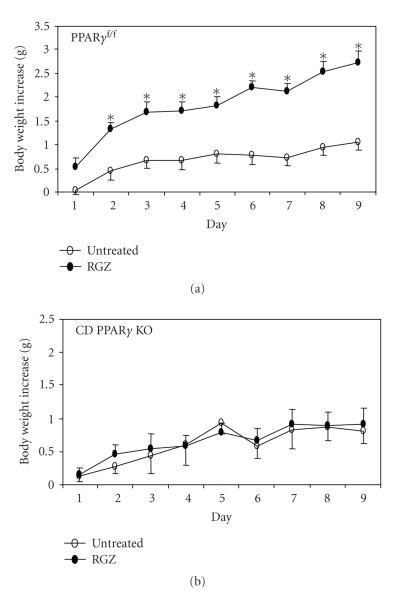
Body weight gains in untreated and rosiglitazone (RGZ)-treated PPAR*γ* ^f/f^ mice (a) and CD PPAR*γ* knockout mice (b) (adapted from [28]).*, *P* < .05 versus vehicle at the corresponding time point.

**Figure 2:**
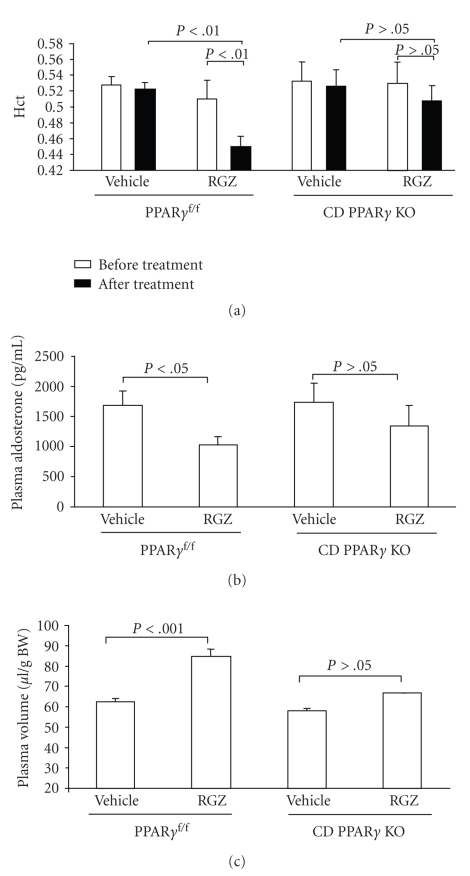
Changes in plasma volume in PPAR*γ* ^f/f^ and CD PPAR*γ* knockout mice following rosiglitazone (RGZ) treatment (adapted from [28]). (a) Hematocrit (Hct) in PPAR*γ* ^f/f^ and CD PPAR*γ* knockout mice before and after RGZ treatment. (b) Plasma aldosterone levels in PPAR*γ* ^f/f^ and CD PPAR*γ* knockout mice following RGZ treatment. (c) Determination of plasma volume in PPAR*γ* ^f/f^ and CD PPAR*γ* KO mice by the Evans blue (EB) dye technique.

The involvement of the distal nephron in TZD-induced fluid retention has been assessed in two independent studies using mice with a collecting duct-specific deletion of PPAR*γ* (CD PPAR*γ* KO) [27, 28]. In both studies, the expression of Cre recombinase was driven by an AQP2 promoter highly specific to the collecting duct. In these two studies, the experimental approaches for assessment of fluid retention were quite different: a combination of hematocrit, plasma aldosterone levels, and Evans blue (EB) dye-based measurement of plasma volume in one study (see Figure 1) [28] and determination of total water content in the other [27]. Remarkably, both studies reported a similar phenotype in that the conditional PPAR*γ* knockout mice proved to be resistant to the rosiglitazone- or pioglitazone-induced body weight gain and plasma volume expansion found in mice expressing PPAR*γ* in the collecting duct. As shown in Figure 1, a nine-day rosiglitazone treatment induced a gradual and significant increase in body weight in floxed mice when compared to untreated floxed controls (2.74 ± 0.25 versus 1.05 ± 0.16 gram, on day 9, *P* < .05). In contrast, body weight gains between rosiglitazone-treated and untreated CD PPAR*γ* KO mice were not significantly different (0.90 ± 0.25 versus 0.81 ± 0.19 gram, on day 9, *P* > .05). Rosiglitazone treatment in the control mice induced plasma volume expansion, which was reflected by a significantly decreased hematocrit and plasma aldosterone levels as well as by a 32.2% increase in plasma volume as assessed by the EB dye technique. In contrast, rosiglitazone-treated CD PPAR*γ* KO mice exhibited nonsignificant trends toward change in these parameters (see Figure 2). These two studies also provided evidence that exposure of primary collecting duct cells to PPAR*γ* ligands leads to increased sodium transport as assessed by measurements of ^22^Na^+^ flux and transepithelial resistance.

Guan et al. examined the effects of pioglitazone on the expression of *α*-, ß-, and *γ*-ENaC subunits in cultured inner medullary collecting duct (IMCD) cells [27]. Notably, within one hour following treatment of IMCDs with pioglitazone (1 *μ*M), *γ*-ENaC mRNA expression increased roughly 10 folds before gradually diminishing. This stimulatory effect appeared to be specific for *γ*-ENaC mRNA, because *α*-ENaC and ß-ENaC mRNA levels did not show any change in response to treatment with pioglitazone. Interestingly, PPAR response elements (PPREs) are identified in intron 1 but not in the 5′ flanking region of the *γ*-ENaC gene. Chromatin immunoprecipitation (ChIP) of genomic DNAisolated from cultured mouse IMCDs revealed a physical interaction between PPAR*γ* and *γ*-ENaC genomic DNA. Somewhat unexpectedly, the PPAR*γ* binding site was shown to be located outside intron 1 of the *γ*-ENaC gene. Overall, these data support *γ*-ENaC as a direct target gene of PPAR*γ* in the collecting duct cells, although the exact mechanism remains to be elucidated.

However, the role of ENaC as a direct target of PPAR*γ* has not always been demonstrable. Nofziger et al. reported that, in collecting duct cell lines, PPAR*γ* agonists failed to enhance basal or insulin-stimulated sodium transport as assessed by measurement of short-circuit current (Isc) [29]. This study also did not find that PPAR*γ*-induced changes in the amount of SGK1 transcript or protein expression. Additionally, there is no solid evidence for major changes in renal expression of any of the ENaC subunits in response to PPAR*γ* ligands in vivo [13, 26, 30]. More recently, Vallon et al. reported that collecting duct-specific gene inactivation of *α*-ENaC in the mouse does not attenuate the rosiglitazone-induced body weight gain [31]. In this study, the Hoxb-7 promoter was used to inactivate *α*-ENaC in the collecting duct, while leaving ENaC expression in the cortical connecting tubule (CNT) intact [32]. As expected, in the floxed control mice, rosiglitazone treatment (320 mg/kg diet) rapidly increased body weight (ΔBW day 11: 4.5 ± 0.8% versus 1.1 ± 0.6%, *P* < .05) and lowered hematocrit (44 ± 1.0% versus 47 ± 1%, *P* < .0005), while rosiglitazone treatment increased body weight (ΔBW: 7.3 ± 0.9% versus 0.9 ± 0.7%, *P* < .0005) and lowered hematocrit (42 ± 2% versus 47 ± 1%, *P* < .05) in *α*-ENaC collecting duct knockout mice. These data may argue against collecting duct ENaC playing a significant role in mediating the adverse effect of rosiglitazone. However, involvement of ENaC activity in the CNT cannot be ruled out. To resolve this issue, AQP2-Cre mice could be used to inactivate ENaC in the entire collecting duct system.

The negative results discussed above prompt consideration of alternative mechanisms for explaining PPAR*γ*-mediated increases in distal tubular fluid reabsorption. There is a significant amiloride-insensitive component in the rosiglitazone-induced increases in sodium transport [28]. The possibility exists that increased reabsorption may occur by way of a paracellular route. For example, PPAR*γ* may regulate the tight junction leading to altered permeability to sodium or other electrolytes. In an in vitromodel of differentiating normal human urothelial (NHU) cells, PPAR*γ* activation in conjunction with epidermal growth factor receptor (EGFR) blockade led to the de novo expression of claudin 3 mRNA and protein and downregulation of claudin 2 transcription [33]. These results suggest a role for PPAR*γ* and EGFR signaling pathways in regulating the tight junction formation in NHU cells. There is an intriguing possibility that a similar mechanism may operate in renal epithelial cells. Another possible mechanism is that PPAR*γ* may regulate transport of ions other than sodium. Further studies are clearly needed to explore not only ENaC-dependent, but also ENaC-independent mechanisms, for TZD-activated fluid reabsorption in the distal nephron.

## 3. VASCULAR MECHANISM

PPAR*γ* is expressed in the vascular system [34], including endothelial cells [35, 36], vascular smooth muscle cells (VSMC) [37] as well as monocyte/macrophages [38, 39]. Several lines of evidence suggest that PPAR*γ* regulates various aspects of vascular function, including capillary permeability. Increased capillary permeability leads to extravasation of fluid and is thought to contribute to edema in patients treated with TZDs. Donnelly et al. were the first to examine the direct effect of rosiglitazone on endothelial barrier function using an in vitro system of pulmonary artery endothelial cell monolayers. Transendothelial albumin flux was measured using EB dye-labeled albumin. They found that exposure to high concentrations of rosiglitazone for four hours increased transendothelial albumin flux dose-dependently, with a noticeable effect at 10 *μ*M and a maximal effect at 100 *μ*M. This hyperpermeability response to high concentrations of rosiglitazone was fully reversible by washing rosiglitazone off the monolayer. After incubation for 24 to 48 hours, the effect of rosiglitazone began to subside. High concentrations of rosiglitazone (0.1–1 mM) are also needed to induce a vasodilator effect in isolated arteries [40]. Future studies, ideally employing gene knockout mice, may determine the extent of PPAR*γ* mediation of the vascular response to high concentrations of TZDs. The mechanism of TZD-induced capillary permeability is not well characterized but may involve a number of factors, notably vascular endothelial growth factor (VEGF), nitric oxide, and protein kinase C, each of which is discussed below.

VEGF is a potent cytokine that augments vascular permeability in tumors, healing wounds, retinopathies, many important inflammatory conditions, and certain physiological processes, such as ovulation and corpus luteum formation [41]. VEGF is estimated to be 50 times more potent than histamine in enhancing vascular permeability [41]. The gene transfer of naked plasmid DNA encoding the 165-amino acid isoform of VEGF in patents with peripheral artery disease causes peripheral edema [42]. Evidence suggests an involvement of VEGF in TZD-induced edema. The study of Emorto et al. was the first to report that plasma levels of VEGF are significantly increased in troglitazone-treated subjects (120.1 ± 135.0 pg/mL) compared with those treated with diet alone (29.2 ± 36.1 pg/mL), sulfonylurea (25.8 ± 22.2 pg/mL), or insulin (24.6 ± 19.0 pg/mL). The effect of troglitazone on increased VEGF levels was further supported by plasma VEGF levels in five patients before treatment (20.2 ± 7.0 pg/mL), after three months of troglitazone treatment (83.6 ± 65.9 pg/mL), and three months after discontinuation (28.0 ± 11.6 pg/mL). These authors further demonstrated that troglitazone, as well as rosiglitazone, at the plasma concentrations observed in patients, increased VEGF mRNA levels in 3T3-L1 adipocytes. The finding suggests that PPAR*γ* activation may directly stimulate expression of VEGF that leads to tissue edema. However, it is puzzling that several other studies show that PPAR*γ* negatively regulates VEGF signaling. In transformed and primary endometrial cells rosiglitazone or 15-deoxy-delta 12,14-prostaglandin J_2_ (15d-PGJ_2_) decreased VEGF protein secretion [43]. In transiently transfected Ishikawa cells, rosiglitazone repressed VEGF gene promoter-luciferase activation with an IC [37] approximately 50 nM. By using truncated and mutated VEGF promoter constructs, this study further revealed that the PPAR*γ*-regulated domain is a direct repeat (DR)-1 motif −443 bp upstream of the transcriptional start site [43]. Similarly, rosiglitazone attenuated VEGF-induced proliferation and migration of human pulmonary valve endothelial cells (HPVECs) [44]. Rosiglitazone also antagonized VEGF-induced nuclear factor translocation in activated T cells subtype c1 (NFATc1) [44]. Furthermore, rosiglitazone markedly decreased VEGF-induced tube formation and cell migration in human umbilical vein endothelial cells [45]. Taking these studies together, it seems likely that PPAR*γ* exerts a dual effect on VEGF signaling, possibly depending on cell type.

Nitric oxide (NO) is a ubiquitous, naturally occurring molecule found in a variety of cell types and organ systems. Endothelial cells are rich in NO, which has been shown to regulate many aspects of vascular function, including vascular permeability. Polikandriotis et al. report that 15d-PGJ_2_ and ciglitazone increase cultured endothelial cell NO release without increasing the expression of endothelial nitric oxide synthase (eNOS) [46]. This study provided further evidence that PPAR*γ* activation leads to eNOS ser1177 phosphorylation [46]. It seems plausible that the stimulation of eNOS-derived NO may contribute to TZD-induced edema. St-Pierre et al. examined the effect of rosiglitazone on muscle vasopermeability and NO system in the fructose-fed rat model [47]. In this study, extravasation of EB dye in vivo in specific muscle groups was used to assess vascular permeability. Fructose-fed rats treated with rosiglitazone had a 30–50% increase in extravasation of EB in the the Rectus femoris, soleus, gastrocnemius lateralis, vastus lateralis, and tibialis cranialis skeletal muscles [47]. In homogenates of skeletal muscles (vastus lateralis) from fructose-fed rats, rosiglitazone resulted in a significant increase in nitric oxide synthase (NOS) activity and eNOS immunoreactive content compared to the control animals [47]. Unexpectedly, the immunoreactive level of the most abundant muscle NOS isoforms, neuronal NOS (nNOS), remained unchanged.

Protein kinase C (PKC) plays a major role in determining vascular permeability through phosphorylation of the cytoskeleton proteins that form the tight intercellular junction [48–51]. In the study of Sotiropoulos et al., rosiglitazone treatment selectively activated PKC in fat and retinal tissues in parallel with the increased vascular permeability in these tissues [52]. The activation of PKC is evaluated by determining the enzyme activity together with tissue levels of diacylglycerol (DAG), a strong PKC activator [52]. These investigators tested the effect of PKC*β* inhibition and gene knockout but did not determine specific PKC isoforms. They found that posttreatment with ruboxistaurin (RBX), a PKC*β* inhibitor, effectively attenuated the increases in capillary permeability, water content, and weight of epididymal fat, as well as the increase in body weight associated with rosiglitazone treatment; this finding was also confirmed by using PKC*β* KO mice [52].

## 4. POTENTIAL THERAPIES

### 4.1. Inhibition of sodium transport in the collecting duct

The use of diuretics for management of TZD-induced fluid retention has been evaluated by several case reports [2, 53] and, recently, by a controlled trial [54]. Most case reports show that the edema is refractory to a loop diuretic (furosemide) and that the symptoms resolve only after discontinuation of TZD. The recent controlled trial involved 381 patients with type 2 diabetes. It examined the effect of three diuretics that act with different mechanisms on rosiglitazone-induced body weight gain and plasma volume [54]. The diuretics included furosemide, which inhibits the Na-K-Cl cotransporter in the thick ascending limb of the loop of Henle, hydrochlorothiazide (HCTZ), which acts to inhibit the Na-Cl cotransporter in the distal convoluted tubule, and spironolactone (SPIRO), which is an ENaC inhibitor in the collecting duct. The degree of fluid retention in this study was evaluated by measuring changes in the hematocrit as an index of changes in plasma volume, body weight, total body water, and extracellular fluid changes determined by noninvasive bioelectrical impedance with an Akern soft tissue analyzer. SPIRO and HCTZ both effectively reduced fluid retention and body weight while furosemide had only a limited effect. The effectiveness of SPIRO may be attributable to the ability of this diuretic to interfere with the sodium retaining action of PPAR*γ* in the collecting duct. It is unclear whether the same mechanism can explain the action of HCTZ. Thiazide diuretics act primarily in the proximal part of the distal convoluted tubules where they inhibit Na^+^/Cl^−^ cotransport [55, 56], but they are also reported to inhibit salt and water reabsorption in the medullary collecting duct [57]. The reason for the lack of diuretic response of TZD-treated diabetics to furosemide is not entirely clear, but one possible explanation might be the lack of distal effect of this loop diuretic. Another possibility is that TZD-induced fluid retention may be associated with impaired transport machinery in the thick ascending limb. Possibly secondary to the volume expansion, the plasma level of atrial natriuretic factor (ANF) is elevated in TZD-treated diabetics [54]. ANF inhibits NaCl reabsorption in the loop of Henle as well as in other sites of nephron through the activation of guanylyl cyclase receptors that release cyclic GMP [58]. It also remains possible that PPAR*γ* may negatively affect NaCl transport in the loop of Henle.

The experimental evidence favoring ENaC as a potential target of PPAR*γ* in the distal nephron seems to provide a rationale for the use of amiloride as a specific ENaC inhibitor for treatment of TZD-induced fluid retention. Unfortunately, amiloride was not included in this clinical trial [54]. In the mouse, pretreatment with amiloride effectively prevents body weight gain and fluid retention produced by pioglitazone. However, in the rat model, posttreatment with amiloride unexpectedly exacerbates the fluid retention induced by farglitazar. It is unclear whether this discrepancy between the studies is due to species differences, PPAR*γ* ligand activity, or the different timing of amiloride treatment.

### 4.2. Combination of a PPAR*γ* and a PPAR*α* agonist

Boden et al. examined the effect of the combined use of rosiglitazone and fenofibrate in patients with type 2 diabetes [59]. Compared with rosiglitazone alone, rosiglitazone/fenofibrate proved significantly more effective in lowering fasting free fatty acid levels and tended to be more effective in achieving plasma glucose control. Interestingly, rosiglitazone/fenofibrate completely prevented the increase in body weight and body water content associated with rosiglitazone. This study is the first to show that the combined use of a PPAR*γ* and a PPAR*α* agonist can prevent rosiglitazone-induced fluid retention. The investigators did not propose a mechanism to explain this phenomenon. The two PPAR isoforms occur in different locations along the nephron. PPAR*α* mRNA is found predominately in the cortex and is specifically localized in the proximal convoluted tubule (PCT). PPAR*γ* is abundant in the renal inner medulla, specifically localized to the inner medullary collecting duct [23, 25]. The difference in nephron localization does not seem to favor the direct interaction between the two PPAR isoforms. However, it remains possible that low PPAR*α* activity in the collecting duct may antagonize the sodium-retaining action of PPAR*γ*. Future studies are needed to investigate whether an interaction occurs in the collecting duct or another location.

Dual PPAR*α*/*γ* agonists have been developed by several pharmaceutical companies, and some have undergone or are currently undergoing clinical trials [60–62]. Unfortunately, muraglitazar, the first dual PPAR*α*/*γ* agonist, has been associated with an excessive incidence of major adverse cardiovascular events, including myocardial infarction, stroke and transient ischemic attack, chronic heart failure and death [62]. This finding raises significant safety concerns about the dual agonists as well as the combination of a PPAR*γ* and a PPAR*α* agonist. In the study of Boden et al., rosiglitazone/fenofibrate appeared to be well tolerated [59]. The safety issues may be related to the ratio of PPAR*γ* to PPAR*α*. The ratios are fixed for the dual agonists, but can be varied by changing the proportion of PPAR*γ* and PPAR*α* agonists. It should be pointed out that Boden's study was limited to a small number of patients and a short period of treatment [59]. The safety issue regarding the combined use of a PPAR*γ* and PPAR*α* agonist needs to be carefully evaluated in larger-scale and longer-term clinical trials as well as animal studies.

### 4.3. Inhibition of protein kinase C

There is functional evidence suggesting the involvement of vascular permeability in TZD-induced body weight gain and fluid retention [52]. Therefore, targeting vascular permeability may provide a potential therapeutic strategy for this side effect of the TZDs. In an animal study, the use of a PKC*β* inhibitor, RBX, to target vascular permeability effectively attenuated the increases in TZD-induced body weight gain [52]. Is there any safety issue related to RBX? In the animal models tested, including Zucker and lean fatty rats, and mice, RBX reduced rosiglitazone-induced capillary permeability, but had no significant effect on the baseline capillary permeability without rosiglitazone treatment. In this short-term animal study, the compound appears to be well tolerated. Another positive note is that RBX is being used in clinical trials for diabetic microvascular complications. In these trials, as well as in animal studies, RBX shows promise for treatment of diabetic retinopathy and nephropathy without noticeable side effects [63, 64].

## 5. CONCLUSIONS

**Figure 3:**
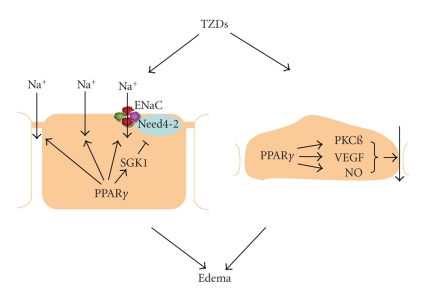
The mechanism for thiazolidinedione- (TZD-) induced edema. In the renal collecting duct, PPAR*γ* activation increases sodium reabsorption through ENaC-dependent and independent mechanisms. In the blood vessels of adipose tissues, PPAR*γ* ligands activate PKCß, VEGF, and NO, which together lead to increased endothelial permeability. The increased renal sodium retention at the level of the collecting duct in conjunction with increased vascular permeability may determine edema development.

The fluid retention and rapid body weight gain induced by TZD treatment are caused by increased fluid reabsorption in the distal nephron as well as increased vascular permeability in adipose tissues (see Figure 3). The molecular mechanisms of the effects of TZDs in renal collecting duct and in blood vessels remain unknown. Despite documentation of ENaC as a molecular target of TZDs in the collecting duct, increasing evidence indicates ENaC-independent mechanisms that may involve changes in paracellular transport. PKCß is shown to mediate TZD-induced vascular permeability in adipose tissues. More studies are required for determination of the signaling pathway responsible for PPAR*γ*-dependent tissue-specific activation of PKCß. Currently, there are no effective therapies for the side effects of TZDs except drug withdrawal. A number of potential treatment strategies that target collecting duct sodium transport (amiloride) and vascular permeability (PKC inhibitors) have been developed from animal studies and should be evaluated by future clinical trials.
